# P06 Antibiotic usage post lower limb amputation in a single UK centre: do we need a targeted national approach?

**DOI:** 10.1093/jacamr/dlae217.010

**Published:** 2025-01-23

**Authors:** Nicola L Maddox, Hannah Burnett, Thomas Hardy, Matthew S Kennedy

**Affiliations:** North Bristol NHS Trust; Royal Devon University Healthcare NHS Foundation Trust; Royal Devon University Healthcare NHS Foundation Trust; Royal Devon University Healthcare NHS Foundation Trust

## Abstract

**Background:**

Over 150 000 lower limb amputations (LLA) occur globally per year with surgical site infection (SSI) being a common complication. There is no clear UK national guidance or consensus regarding antibiotic usage post LLA and the extent of the problem is unknown since notification of vascular SSIs to the UK Health Security Agency is not compulsory. In our centre, co-amoxiclav was adopted as post-operative LLA prophylaxis for patients without penicillin allergy.

**Objectives:**

As part of antimicrobial stewardship activities, we aimed to reduce broad spectrum antibiotic usage. We present a data set detailing antibiotic usage post LLA; specifically focusing upon subsequent SSIs and *Clostridioides difficile* infection in patients receiving post-operative prophylactic antibiotics.

**Methods:**

We retrospectively analysed all LLA from September 2020 to October 2023. A total of 132 patient records were identified from EPIC. We surveyed UK trusts to identify their antibiotic practice post LLA with 14 responses.

**Results:**

Overall, 91% (*n*=120) received antibiotics post-operatively. There was a wide variety of antimicrobials and course lengths; the majority were prescribed co-amoxiclav (54%, *n*=65) and received a course length of 1–5 days (52%, *n*=62). Thirty-six percent (*n*=20) had an SSI of which 95% (*n*=19) received post-operative antibiotics. Eighty-five percent (*n*=17) had diabetes mellitus. Fifty-five percent (*n*=11) returned to theatre and one patient (who received antibiotics) died. A total of 4.5% (*n*=6) acquired *C. difficile* infection; 100% had antibiotics post-operatively with an average course length of 5.3 days prior to laboratory diagnosis. Sixty-six percent (*n*=4) received co-amoxiclav. The overall all-cause mortality was 24%; 5 died during admission and 26 died post-admission. Table 1 provides a detailed breakdown of antibiotic lengths and outcomes. Table 2 details pre-operative and post-operative LLA antibiotic guidance in 14 UK trusts.

**Table 1.** Detailed breakdown of antibiotic lengths and outcomes

	No antibiotics	Antibiotics for 24 h (2 doses or 1 day)	Antibiotics for 1–5 days	Antibiotics >5 days	Antibiotics >7 days
Total patients, *n*	12	24	63	6	27
Surgical site infection, % (*n*)	8.3 (1)	25 (6)	11 (7)	33.3 (2)	14.8 (4)
Return to theatre, %	0	12.5 (3)	14 (9)	33.3 (2)	14.8 (4)
*C. difficile*, %	0	8.3 (2)	4.7 (3)	0	3.7 (1)

**Table 2.** Pre-operative and post-operative LLA antibiotic guidance in 14 UK trusts

Trust name	Pre-operative antibiotic prophylaxis	Post-operative antibiotic prophylaxis
University Hospitals Birmingham	Co-amoxiclav	Co-amoxiclav 48 h then wound review
Royal Free, London	Teicoplanin + gentamicin + metronidazole	Metronidazole for 5 days
Leeds Teaching Hospitals	Flucloxacillin + metronidazole + gentamicin for first 24 h	Doxycycline 100 mg BD PO + metronidazole 400 mg TDS PO for maximum 4 days
Royal National Orthopaedic Hospital	Teicoplanin + gentamicin + metronidazole	No further antibiotics
United Lincolnshire Hospitals	Co-amoxiclav	Co-amoxiclav for 5 days if following major trauma No further antibiotics if planned/diabetes, etc.
Gloucestershire	Flucloxacillin + metronidazole + gentamicin for 24 h	No further antibiotics
University Hospitals Plymouth	Co-amoxiclav for 24 h	No further antibiotics
University Hospitals Bristol & Western	Co-amoxiclav	No further antibiotics unless there is residual infected tissue
Hull University Teaching Hospitals	Flucloxacillin + gentamicin + metronidazole	Doxycycline 100 mg BD PO + metronidazole 400 mg TDS PO for 5 days
Portsmouth Hospitals University	Flucloxacillin 1 g IV	No further antibiotics
East Sussex Healthcare	Benzylpenicillin IV	Benzylpenicillin for 5 days or metronidazole 400 mg TDS for 5 days if oral
Kettering GH		None
Bedford		Gentamicin, teicoplanin, metronidazole for 5 days
Northampton GH		Co-amoxiclav for 5 days

**Conclusions:**

We have demonstrated the lack of standardized UK antimicrobial practice post LLA. Our data set enabled greater understanding of antimicrobial stewardship within LLA surgery and subsequently, we implemented formal post LLA guidance for antimicrobials; incorporating a more rational approach whilst minimizing the risk of *C. difficile* infection and continuing effective SSI cover. We will re-audit in the future to assess the effectiveness of this change.

